# Foregut Duplication Cyst of the Stomach: A Case Report and Review of the Literature

**DOI:** 10.1155/2016/7318256

**Published:** 2016-02-21

**Authors:** Y. Tjendra, K. Lyapichev, J. Henderson, C. P. Rojas

**Affiliations:** ^1^Department of Pathology and Laboratory Medicine, Miller School of Medicine, University of Miami, Miami, FL 33136, USA; ^2^Department of Radiology, Miller School of Medicine, University of Miami, Miami, FL 33136, USA

## Abstract

Duplication cyst of the stomach is a rare congenital malformation, typically diagnosed in the first year of life. In most adult cases the cyst remains asymptomatic, but patients may present with abdominal symptoms including epigastric discomfort or pain. We present a case of a 65-year-old male with an asymptomatic gastric tumor diagnosed incidentally during initial workup of his esophageal adenocarcinoma. Computed tomography revealed a low density soft tissue tumor near the gastroesophageal junction. Endoscopic ultrasonography demonstrated a cystic lesion as a hypoechoic round mass with well-defined borders. Following complete laparoscopic resection, microscopic review revealed a cyst lined with respiratory pseudostratified ciliated columnar epithelium and layers of smooth muscle with an outermost thin fibrous capsule consistent with a foregut duplication cyst.

## 1. Introduction

Foregut duplication cyst (FDC) is a rare congenital malformation which may occur at any level of the alimentary tract, with most cases diagnosed during the first year of life [1]. Duplication cysts can occur along with other congenital anomalies, such as annular or heterotopic pancreas, or vertebral anomalies such as spina bifida [2]. The clinical presentation may include a palpable abdominal mass, epigastric pain, or bleeding, but an FDC may remain completely asymptomatic and can be an incidental finding during diagnostic workup for an unrelated cause. Radiologic diagnostic tests are useful for characterization, but an accurate diagnosis can only be rendered by surgical resection and surgical pathology review.

## 2. Clinical Presentation

A 65-year-old male with known history of gastroesophageal reflux disease (GERD) and newly diagnosed esophageal adenocarcinoma was referred to our institution for further management. The patient stated progressive dysphagia with associated significant weight loss without hematemesis, melena, or hematochezia. At the outside institution, computed tomography (CT) and endoscopic ultrasonography (EUS) were performed as part of staging. The results demonstrated a T3 N1 Mx disease which prompted neoadjuvant chemotherapy.

**Figure 1:**
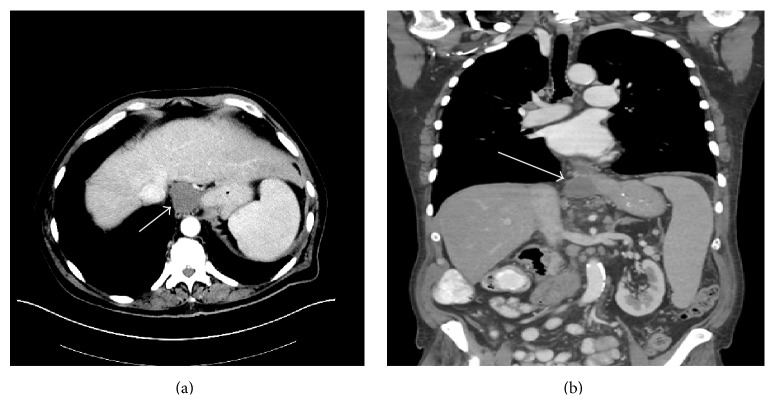
(a) Intravenous contrast enhanced axial CT scan of the abdomen demonstrates a well-circumscribed, low-attenuating, nonenhancing mass (arrow) adjacent to the gastroesophageal junction representing an enteric duplication cyst. (b) Coronal reformat of the intravenous contrast enhanced CT scan of the abdomen also demonstrates a well-circumscribed, low-attenuating, nonenhancing mass (arrow) adjacent to the gastroesophageal junction.

In addition to the adenocarcinoma, CT demonstrated a 4.3 × 4.2 cm low density soft tissue mass at the epigastric region, near the hiatus and the gastroesophageal (GE) junction (Figure 1). EUS showed a 3.6 × 3.0 cm hypoechoic round mass with well-defined borders in the subhepatic peritoneal space along the gastrohepatic region (at the crura). No evidence of invasion was identified. Fine needle aspiration (FNA) was performed and revealed fluid with cells of epithelial origin, and malignancy could not be excluded at this point. The preoperative differential diagnosis included gastrointestinal stromal tumor (GIST) and duplication cyst of the stomach.

No significant reduction of this mass was noted on comparative imaging (CT and EUS) following neoadjuvant chemotherapy. The patient underwent transhiatal esophagectomy and partial gastrectomy, which were followed by an uneventful recovery.

## 3. Pathologic Findings

**Figure 2:**
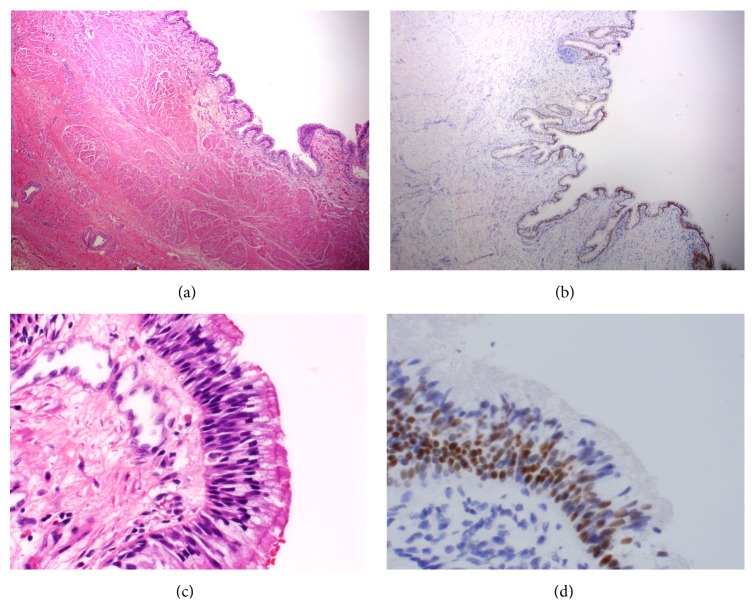
(a) Cystic lesion lined by pseudostratified ciliated columnar epithelium with longitudinal and circular smooth muscle layers (10x). (b) TTF-1 immunohistochemistry highlights the lung type II pneumocytes (10x). (c) High magnification of the pseudostratified ciliate columnar epithelium (60x). (d) High magnification highlights the nuclear staining of TTF-1 (60x).

The specimen consisted of an 11.0 cm segment of esophagus and an 8.0 cm portion of stomach. Macroscopically, 1.0 cm inferior to the GE-junction, a well-circumscribed, white tan, saccular structure measuring 4.0 × 3.4 × 1.2 cm was identified. The cystic lesion was located in the serosa and did not communicate with the gastric lumen. Histologically, it consisted of mucosa lined with respiratory pseudostratified ciliated columnar epithelium (PCCE), which stained positive for thyroid transcription factor 1 (TTF-1) by immunohistochemistry (Figure 2). These findings were consistent with FDC of the stomach.

## 4. Discussion

Cystic lesions of the stomach are rare and, as a result of embryologic anomalies, are of posttraumatic, infectious, or neoplastic origin. They may occur as single or multiple lesions and may appear at any histologic layer of the stomach.

Gastrointestinal malformations occur in the early embryonal stages, and a duplication of the alimentary tract is considered to be a result of abnormal canalization. It may form into a communicating or a noncommunicating cystic or tubular structure, which is lined by a mucosal membrane [3]. These duplications are postulated to be formed before differentiation of epithelium into the characteristic adult types. Thus, they are generally named after the organ they are associated with rather than the mucosa lining them [4]. Duplication cysts can occur at any level of the alimentary tract, but the most common location is the ileum, followed by esophagus, jejunum, colon, stomach, and appendix [5]. Gastric duplication cysts represent about 2–8% of duplication cysts and are mostly located along the greater curvature, adjacent to the gastric wall, because these duplications are usually found to be distributed dorsally to the primitive gut during development [6]. Gastric duplication cysts are located in the lesser curvature in 5.5% of reported cases, and the mechanism by which they develop is not well understood [7].

In the case presented here, the location of the duplication cyst implies the term “gastric duplication cyst,” but the term “foregut duplication cyst” is preferred, as it is lined exclusively with PCCE rather than gastric or intestinal mucosa [3, 8]. To our knowledge, only 23 cases of foregut duplication cyst lined with PCCE have been reported, with the first case published in 1966 [9].

The histogenesis of FDC lined with PCCE remains unclear. Gensler et al. suggested that an FDC lined with PCCE may represent the caudal-most portion of the laryngotracheal outgrowth, which has remained attached to the portion of the primitive foregut destined to become the stomach [9]. This suggests that the foregut duplication cyst in the present case may have arisen as a detached outpouching of the primitive foregut [7].

EUS, CT, and magnetic resonance imaging (MRI) are popular diagnostic methods for the examination of lesions along the gastrointestinal tract. Although FDC are uncommon, EUS findings of gastric duplication cysts have accumulated, and, thus, EUS has proven to be very useful for preoperative diagnosis along with CT or MRI [10].

The clinical presentation of FDC includes abdominal symptoms with a palpable abdominal mass, pain, vomiting, hematemesis, and weight loss [1, 4]. Other symptoms reported are epigastric discomfort or pain and persistent cough with hemoptysis [3, 6, 7, 9]. Most cases in adults are asymptomatic and diagnosed incidentally [11, 12]. In the present case, the FDC was an incidental finding, as the patient did not have any associated symptoms, and it was discovered as part of the presurgical diagnostic workup for his unrelated esophageal carcinoma. Malignant transformation of an FDC is extremely rare. Depending on the epithelial lining of the cyst, several cases of adenocarcinoma, squamous cell carcinoma or neuroendocrine carcinoma, have been reported [13–16].

Even though FDC are rare, they are potentially dangerous; therefore, complete surgical resection is advised whenever possible as either open surgery or minimally invasive laparoscopic or endoscopic surgery (cystectomy, proximal or distal partial gastric resection, or total gastrectomy) [1, 10]. Surgical complications are related to the size and location of the duplication, communication with the gastrointestinal tract or vertebral canal, presence of heterotopic gastric mucosa, and involvement of mesenteric vessels [2].

In summary, FDC of the stomach constitutes a congenital disease whose diagnosis is rendered by microscopic examination and which should be included in the differential diagnosis of a gastric mass [12]. Because of the potential severe complications associated with FDC including perforation, bleeding, possible fistula formation and obstruction, and its potential for malignant transformation, the first line therapy is complete surgical resection.
